# Path tracking control of a steerable catheter in transcatheter cardiology interventions

**DOI:** 10.1007/s11548-024-03069-3

**Published:** 2024-02-22

**Authors:** Xiu Zhang, Aditya Sridhar, Xuan Thao Ha, Syed Zain Mehdi, Andrea Fortuna, Mattia Magro, Angela Peloso, Anna Bicchi, Mouloud Ourak, Andrea Aliverti, Emiliano Votta, Emmanuel Vander Poorten, Elena De Momi

**Affiliations:** 1 Department of Electronics, Information and Bioengineering, Politecnico di Milano, 20133 Milan, Italy; 2 Department of Mechanical Engineering, KU Leuven, 3001 Leuven, Belgium

**Keywords:** Cardiac interventions, Catheter robot, Motion control, EM sensor, FBG-based sensors

## Abstract

****Purpose**:**

Intracardiac transcatheter interventions allow for reducing trauma and hospitalization stays as compared to standard surgery. In the treatment of mitral regurgitation, the most widely adopted transcatheter approach consists in deploying a clip on the mitral valve leaflets by means of a catheter that is run through veins from a peripheral access to the left atrium. However, precise manipulation of the catheter from outside the body while copying with the path constraints imposed by the vessels remains challenging.

****Methods**:**

We proposed a path tracking control framework that provides adequate motion commands to the robotic steerable catheter for autonomous navigation through vascular lumens. The proposed work implements a catheter kinematic model featuring nonholonomic constraints. Relying on the real-time measurements from an electromagnetic sensor and a fiber Bragg grating sensor, a two-level feedback controller was designed to control the catheter.

****Results**:**

The proposed method was tested in a patient-specific vessel phantom. A median position error between the center line of the vessel and the catheter tip trajectory was found to be below 2 mm, with a maximum error below 3 mm. Statistical testing confirmed that the performance of the proposed method exhibited no significant difference in both free space and the contact region.

****Conclusion**:**

The preliminary in vitro studies presented in this paper showed promising accuracy in navigating the catheter within the vessel. The proposed approach enables autonomous control of a steerable catheter for transcatheter cardiology interventions without the request of calibrating the intuitive parameters or acquiring a training dataset.

## Introduction

Mitral regurgitation (MR) is a type of heart valve disease in which the valve between the left atrium (LA) and the left ventricle (LV) does not completely close, allowing blood to leak backward. MR increases the pressure in the pulmonary venous channel and the left atrial chamber, weakening the heart walls, causing shortness of breath, fatigue, and in chronic cases, heart failure. According to the report on the population-based studies in USA, 1.7% of adult population and 9.3% of adults over the age of 75 suffer from MR [1]. Moreover, the annual mortality rate is about 34%. Open-chest surgery can provide immediate relief unlike medication, but 50 % of the MR patients are not recommended open-heart surgery due to their age and possibilities of postoperative complications [2].

**Fig. 1:**
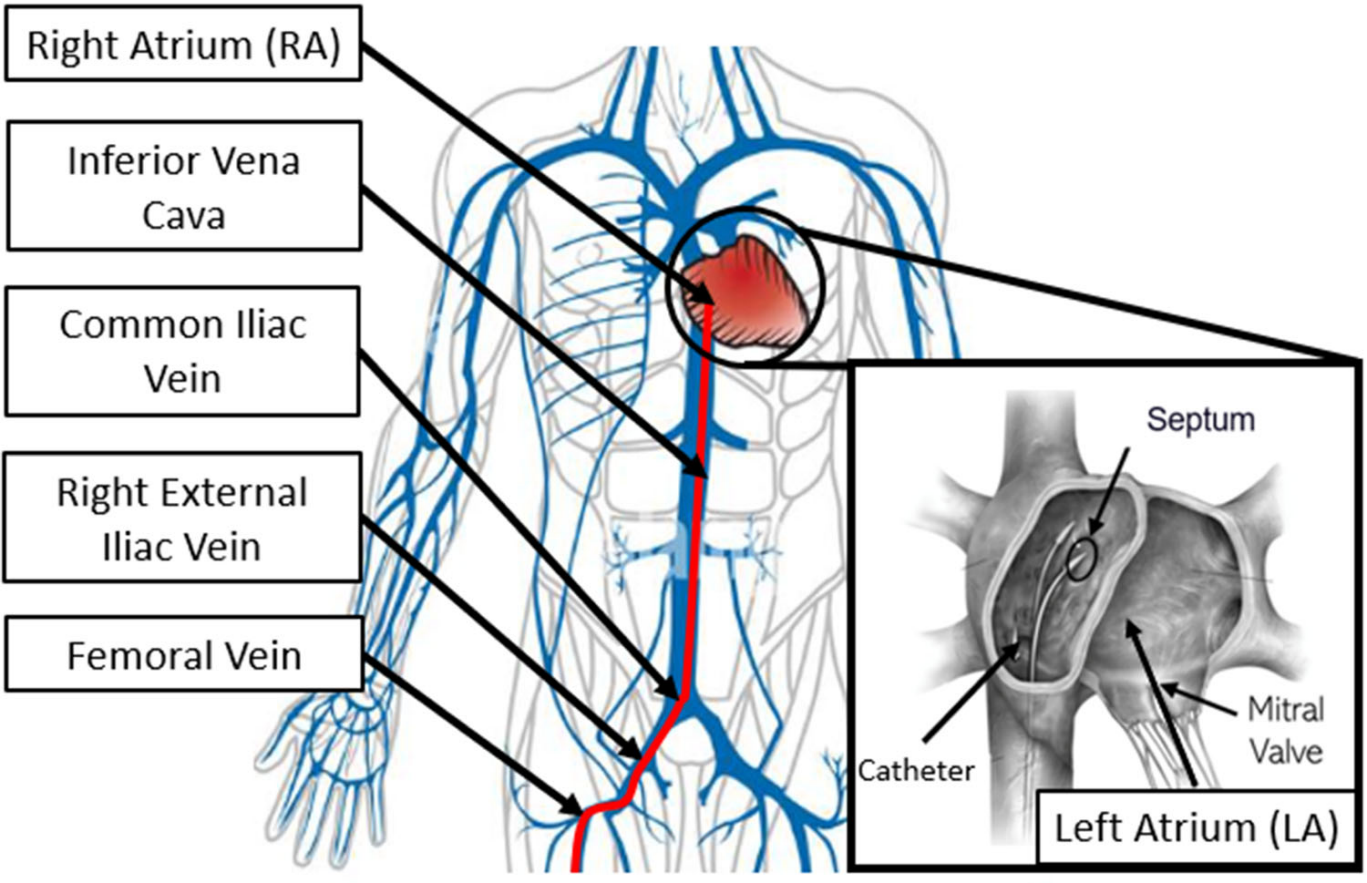
Path of the transcatheter mitral valve repair procedure: The catheter passes through the femoral vein, right external iliac vein, common iliac vein, and inferior vena cava and arrives at the right atrium. From there, the mitral valve is reached by a transseptal puncture

Transcatheter mitral valve repair approaches are gaining popularity due to reduced invasiveness and shorter recovery time. Moreover, transcatheter approaches offer an alternative treatment for patients who cannot undergo open-chest surgery. With systems such as the MitraClip^TM^ (MC) device (Abbott Laboratories, IL), mitral valve competence can be restored by enforcing leaflet coaptation through the implantation of a clip. The cardiologist inserts a steerable catheter, commonly called sheath catheter, from the femoral vein, passing a sequence of narrow and rugged vessels to reach the right atrium (RA). From there, the LA is accessed through a puncture in the atrial septum (Fig. 1). After successfully accessing the LA, the operator inserts the delivery catheter through the sheath catheter to the mitral valve and implants the clip [3]. However, to visualize the catheter during the procedure, both patients and operators would be exposed to damaging radiation [4]. In addition, given the poor image quality and the lack of depth, a significant risk of embolization or perforation exists [5].

To address these challenges in maneuvering the MC steerable sheath catheter, we proposed a control framework for a customized-built actuator to autonomously advance it in a continuous fashion along a pre-planned path. By providing the appropriate steering commands based on the catheter kinematic model, the tip of the catheter tracks the center line of the vessel to avoid intense contact between the acute catheter tip and the fragile vessel wall. To ensure precise steering, a feedback controller is implemented to control the tension on the tendon and reject the error of the steering angle in the joint space. Experiments were carried out in a vessel phantom to evaluate the performance of the system.

## Related work

Robotic technology has emerged as an important tool for catheters deployment. In 2011, CorPath 200 (Corindus Inc., USA) was introduced as the first robot-assisted system to control coronary guidewires and stents for coronary angioplasty procedure [6]. Subsequently, the Sensei Robotic Navigation System (Hansen Medical Inc., USA) was developed and evaluated for catheter ablation of atrial fibrillation [7] and ventricular arrhythmias [8]. The most recent Sensei X Robotic System (Hansen Medical Inc., USA) expands its application on collecting electrophysiological data inside the heart chambers [9]. Furthermore, researchers have developed catheter prototypes with haptic interfaces to enhance the safety of robot-assisted percutaneous coronary interventions (PCI) [10–12]. Despite successful clinical studies, robot-assisted PCI is only limited to a few clinics with skilled surgeons because of the steep learning curve and expensive hardware [13].

The adoption of task autonomy, where the surgeon supervises the procedure while the robot performs the task autonomously, could address this challenge [14]. Fagogenis et al. contributed to the field by autonomously navigating a concentric tube robot inside the beating heart [15]. Sganga proposed an autonomous navigation method for flexible robots in the lungs to diagnose cancer [16]. Yang et al. introduced an autonomous control algorithm for magnetic microrobot navigation in a large workspace [17]. Although robotic catheters are considered ideal for medical applications due to their particular structure, their compliance can pose difficulties in tracking a constrained path inside the body.

Open-loop control relies on model inversion to determine the appropriate actuation values for achieving the desired robot state. In the context of controlling robotic catheters, some researchers have proposed approaches based on specific kinematic models. [18–20]. Greigarn et al. introduce the pseudo-rigid-body (PRB) model on a robotic catheter by approximating the catheter as rigid links connected by flexible joints [21]. Bailly et al. proposed a differential model-based control scheme for a continuum robot [22]. Although these methods could control catheters in free space, it is still difficult to reach precise motion in realistic scenarios where catheters move in a constrained environment (i.e. the vessels) where contact with the vessel wall is inevitable with open-loop approaches [23].

The data-driven approaches were investigated to overcome the challenges posed by the high complexity of continuum robot kinematics, Michael et al. proposed a model-less control method for controlling a tendon-driven continuum manipulator [24]. In addition, Di et al. introduced a deep learning-based compliant motion controller for a robotic catheter [25]. However, the low compatibility rate of change in the environment and disturbances, as well as the complexity of learning approaches, may limit their application in medical scenarios where surgical instruments are single use and patients have different anatomies [26].

**Fig. 2:**
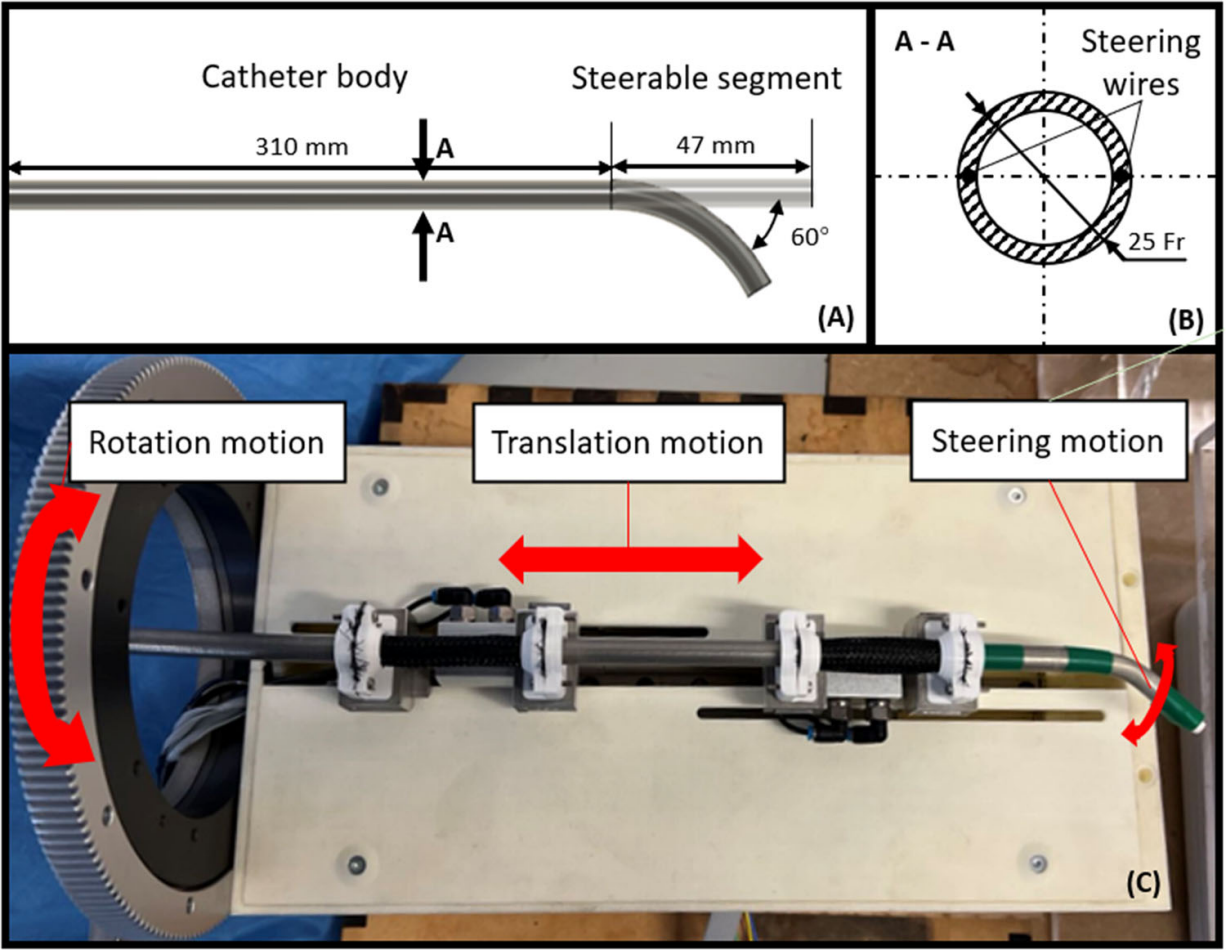
Schematic of the catheter actuation system: **A** top view of the steerable catheter shows a 310-mm-long catheter body and a 47-mm-long steerable segment with a maximum steering angle of $60^{\circ }$; **B** cross-section view of the steerable catheter indicates the outer diameter of the catheter and the position of the steering wires; **C** the robotic system has three actuated DOFs in total, including the rotational motion, the translational motion, and the steering motion

The development of pose-tracking techniques and shape sensors have demonstrated great potential to close the control loop of robotic catheters and compensate for łinaccuracies of the model. In particular, electromagnetic (EM) tracking techniques have been widely applied to track the robotic catheter within the human body [27, 28]. Loschak et al. developed a robotic ultrasound imaging catheter to control the position and orientation based on EM [29]. Omisore et al. proposed a robotic catheter with adaptive compensation of backlash with an EM sensor fixed at the tip of the catheter [30]. In addition, relying on the shape reconstruction from fiber Bragg grating (FBG) sensor, Sefati et al. designed an optimization-based control algorithm to position the continuum manipulators interacting with unknown obstacles [31].

## Method

### Steerable catheter kinematics

The tendon-driven steerable catheter is composed by a 310-mm-long catheter body and a 47-mm-long steerable segment at the distal tip side space(Fig. 2A). The steerable segment is able to generate a $60^{\circ }$ steering angle from the straight position. Two antagonistic steering wires travel along the length of the catheter body up to the tip of the 25-Fr steerable catheter to actuate the steerable segment (Fig. 2B). Expect for the tendon-driven steering motion, based on our previous work [32], a catheter driver system with the sleeve-based grippers and the spur gear is used to generate decoupled 2 degrees of freedom (DOFs) (i.e., translation and rotation) (Fig. 2C).

**Fig. 3:**
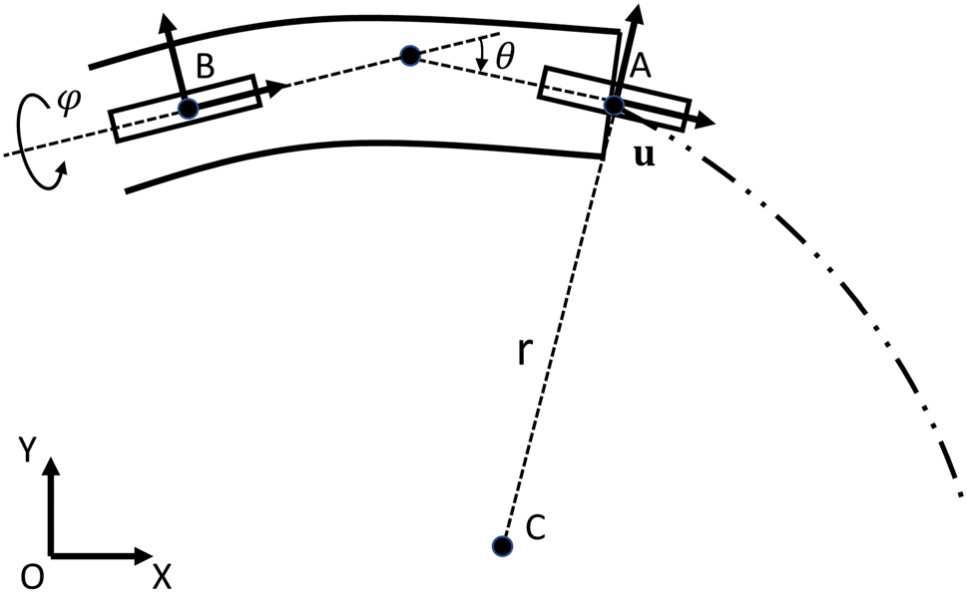
Non-holonomic motion of the catheter modeled with a so-called bicycle model showing the steerable segment of the catheter showing the front and rear “wheels” at frame A and frame B of a bicycle model, including three DOFs: rotation angle ${\varvec{{\varphi }}}$, insertion speed $\textbf{u}$, and in-plane steering angle $\theta$

The “bicycle” model is often used in autonomous driving by characterizing the system with two main wheels, capturing both lateral and longitudinal motion. Regarding the catheter, assuming that the translational motion and the rotational motion propagate ideally from the base through the catheter body to the tip, the distal steerable segment of the catheter can be modeled as a kinematic nonholonomic system, an extension of the “bicycle" model [33], including all the three actuated DOFs. As shown in Fig. 3, the front wheel (A) of the bicycle is attached to the tip of the catheter, and the rear wheel (B) is located on the proximal end of the steerable segment. Generally speaking, advancing the catheter can be approximated as “cycling” forward at a speed, $\textbf{u}$, and bending the catheter with an angle, $\theta$, is like steering the front wheel of the bicycle. The combination of these two motions will generate a circular trajectory with a constant curvature radius, r, and a rotation center at point C. Furthermore, by rotating the catheter about its longitudinal axis with a rotation angle, $\varphi$, one can control the angle of the planar trajectory.

### Control strategy

**Fig. 4:**
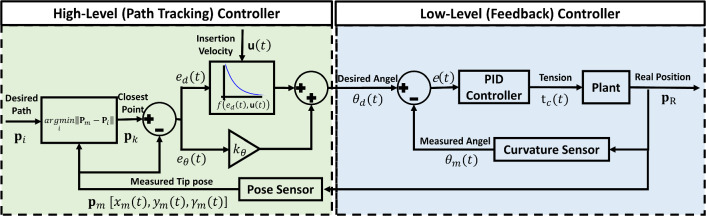
Control framework: The high-level controller accepts the preoperative desired path $\textbf{p}_i$ as input and finds the closest point $\textbf{p}_k$ with respect to the measured tip pose $\textbf{p}_m [ x(t)_m,y(t)_m,\gamma (t)_m ]$ from the pose sensor. Then it computes the distance error $e_d(t)$ and the orientation error $e_\theta (t)$ between $\textbf{p}_k$ and $\textbf{p}_m$. Combining the insertion velocity $\textbf{u}(t)$ in the nonlinear part and the orientation error $e_\theta (t)$ with the orientation gain $k_\theta$, the path tracking controller outputs the desired angle $\theta _d(t)$ to the steering controller. On the low-level side, the PID feedback controller obtains the measured angle $\theta _m(t)$ from the curvature sensor and outputs the tension on the tendon $\textrm{t} _c(t)$ to the plant

This work combines two control strategies: 1) path tracking control, a high-level controller, is used to correct the motion of the steerable catheter when it deviates from the path; 2) feedback control, a low-level controller, is used to drive the actuation system to reach a desired steering angle (Fig. 4).

#### High-level (Path tracking) controller

**Fig. 5:**
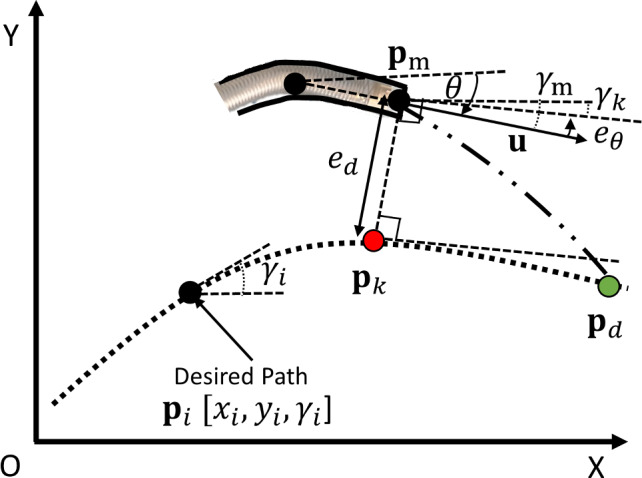
Path tracking strategy: based on the current position $\textbf{p}_m$ and the desired path $\textbf{p}_i [x_i,y_i,\gamma _i]$, the steering angle $\theta$ can be computed by considering the distance error $e_{d}$ and the orientation error $e_{\theta }$ with respect to the closed point $\textbf{p}_k$ on the desired path. At the next time step, the catheter will intersect with the path on point $\textbf{p}_d$ with a steady speed $\textbf{u}$

The path tracking controller is a nonlinear feedback controller which reduces the tracking error between the measured tip position $\textbf{p}_{\textrm{m}}$ and the closest point $\textbf{p}_k$ on the desired path $\textbf{p}_i [ x_i,y_i,\gamma _i]$. The control law consists of two parts, which account for the orientation error, $e_{\theta } \left( t \right)$, and the distance offset error, $e_{\delta } \left( t \right)$, as shown in Eqs. (1), (2), and (3). These two terms are the steering control elements based on the “Stanly method” [34]. Within the control loop, $e_{\theta } \left( t \right)$ is intuitively aligning the orientation of the tip, $\gamma _m \left( t \right)$, to match the orientation of the desired path, $\gamma _k\left( t \right)$, and the second term adjusts the steering angle in (nonlinear) proportion to the distance error $e_{d}\left( t \right)$. In other words, it controls the steering angle, $\theta _{d} (t)$, such that the intended trajectory intersects the path at point $\textbf{p}_d$ at the next time step $t+1$ (Fig. 5). Additionally, the contribution of $e_{\theta } \left( t \right)$ and $e_{\delta } \left( t \right)$ can be adjusted by tuning the orientation gain factor, $k_{\theta }$, for adapting to different paths. The steering gain factor, $k_{d}$, determines the rate of convergence toward the path.

1 $$\begin{aligned} \theta _{d}\left( t \right)= & {} k_{\theta } e_{\theta } \left( t \right) \pm e_{\delta } \left( t \right) \end{aligned}$$

2 $$\begin{aligned} e_{\theta } \left( t \right)= & {} \gamma _{\textrm{m}} \left( t \right) - \gamma _k\left( t \right) \end{aligned}$$

3 $$\begin{aligned} e_{\delta } \left( t \right)= & {} f \left( e_{d}\left( t\right) , \textbf{u} \left( t \right) \right) = \arctan \left( \frac{k_{d}e_{d}\left( t\right) }{\left\| \textbf{u} \left( t \right) \right\| } \right) \end{aligned}$$

According to the bicycle model [35], the time derivative of the distance error, $\dot{e}_{d}\left( t \right)$, can be written as follows:

4 $$\begin{aligned} \dot{e}_{d}\! \left( t \right) \!=\!-\!\left\| \textbf{u} \left( t \right) \right\| \!\sin \!\left( \!\arctan \!\left( \!\frac{\!k_{d}e_{d}\!\left( t\right) }{\left\| \textbf{u} \left( t \right) \right\| ) } \!\right) \!\right) \!=\!\frac{-k_{d}e_{d}\left( t\right) }{\sqrt{1\!+\!\left( \frac{k_{d}e_{d}\left( t\right) }{\left\| \textbf{u} \left( t \right) \right\| } \right) ^{2} } } \end{aligned}$$

and hence, for a small distance error $$ e_{d }$$,

5 $$\begin{aligned} e_{d } \left( t \right) \approx e_{d } \left( 0 \right) exp-k_{d}t \end{aligned}$$

Thus, the distance error converges exponentially to zero.

#### Low-level (feedback) controller

The desired steering angle is then sent to the low-level controller, which is a PID feedback controller in the actuation space (Eq. (6)).

6 $$\begin{aligned} t_C(t) = K_p\cdot e(t) + {K_i\cdot dt}\sum _{i=0}^{t}e(t) +K_d\frac{e(t) - e(t-1)}{dt} \end{aligned}$$

where $t_C(t)$ is the tension applied on the tendon, *e*(*t*) is the error between the desired steering angle, $\theta _d(t)$, and the measured steering angle, $\theta _m(t)$, and $K_p, K_i, K_d$ are the proportional, integral and derivative gains, respectively.

## Experimental setup and protocols

### Experimental setup

**Fig. 6:**
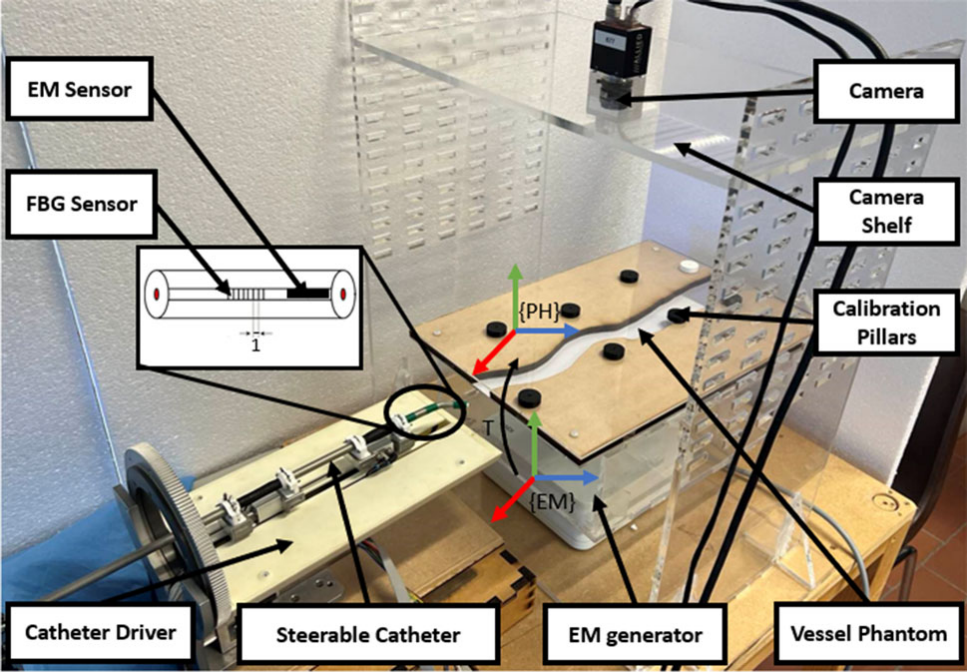
Experimental setup: A tendon-driven steerable catheter, equipped with an EM sensor and a FBG style of 1 cm spacing. The catheter drive advances the steerable catheter into the vessel phantom, which is placed on top of the EM generator. In addition, eight calibration pillars are used to find the transformation matrix $\textbf{T}$, between the EM reference frame $\{\textbf{EM}\}$, and the phantom reference frame $\{\textbf{PH}\}$. A camera is mounted above to record the trajectory as the ground truth

The experiment was performed by actuating and controlling a tendon-driven steerable catheter using a sleeve-based robotic catheter driver. Moreover, a 6-DOFs EM sensor (NDI, Canada) was attached to the tip of the steerable catheter as the pose sensor, and an FBG stylet (FBGS, Technologies GmbH. Germany) with 1 cm spacing was placed in the channel of the catheter as the curvature sensor (Fig. 6). All the data communication was conducted and synchronized on the ROS (robot operating system).

The EM generator was placed below the catheter to generate the magnetic field for tracking the EM sensor, which acquires the pose at 40 Hz with a standard deviation of 1.4 mm, A calibration procedure was implemented to compute the registration matrix between the vessel phantom and the EM coordinate. The position of eight calibration pillars was measured, which were pre-set on the phantom with a 6-DOFs EM probe (NDI, Canada). Each position was acquired ten times to reduce the acquisition error and reject the noise from the EM sensor. Then the transformation matrix, $\textbf{T}$, between the pillar’s positions in phantom space $\{\textbf{PH}\}$ and the pillar position in the EM space $\{\textbf{EM}\}$ was calculated based on the singular value decomposition approach [36].

The wavelengths from the FBG interrogator Were obtained (FBGS, Technologies GmbH. Germany) and converted to a point cloud depicting the shape of the catheter [37]. To measure the steering angle, two points were selected at the distal end of the FBG stylet to represent the orientation of the tip and two points behind to represent the orientation of the base. Those last two points were selected by calibrating the steering angle with a protractor.

As the ground truth to evaluate the accuracy of the proposed approach, a camera (Prosilica GT, Allied Vision Technologies GmbH. Germany) with a resolution of 31 megapixels and frame rates of 53 frames per second was mounted on a shelf above the phantom to record the trajectory of the catheter (Fig. 6). One piece of green tape was attached to the tip of the catheter as the tracking markers. Before each test, a checkboard with $25\times 25$ mm squares was put below the camera for calibration. The camera data was processed by converting the unit from pixel to millimeter based on the calibration data.

**Fig. 7:**
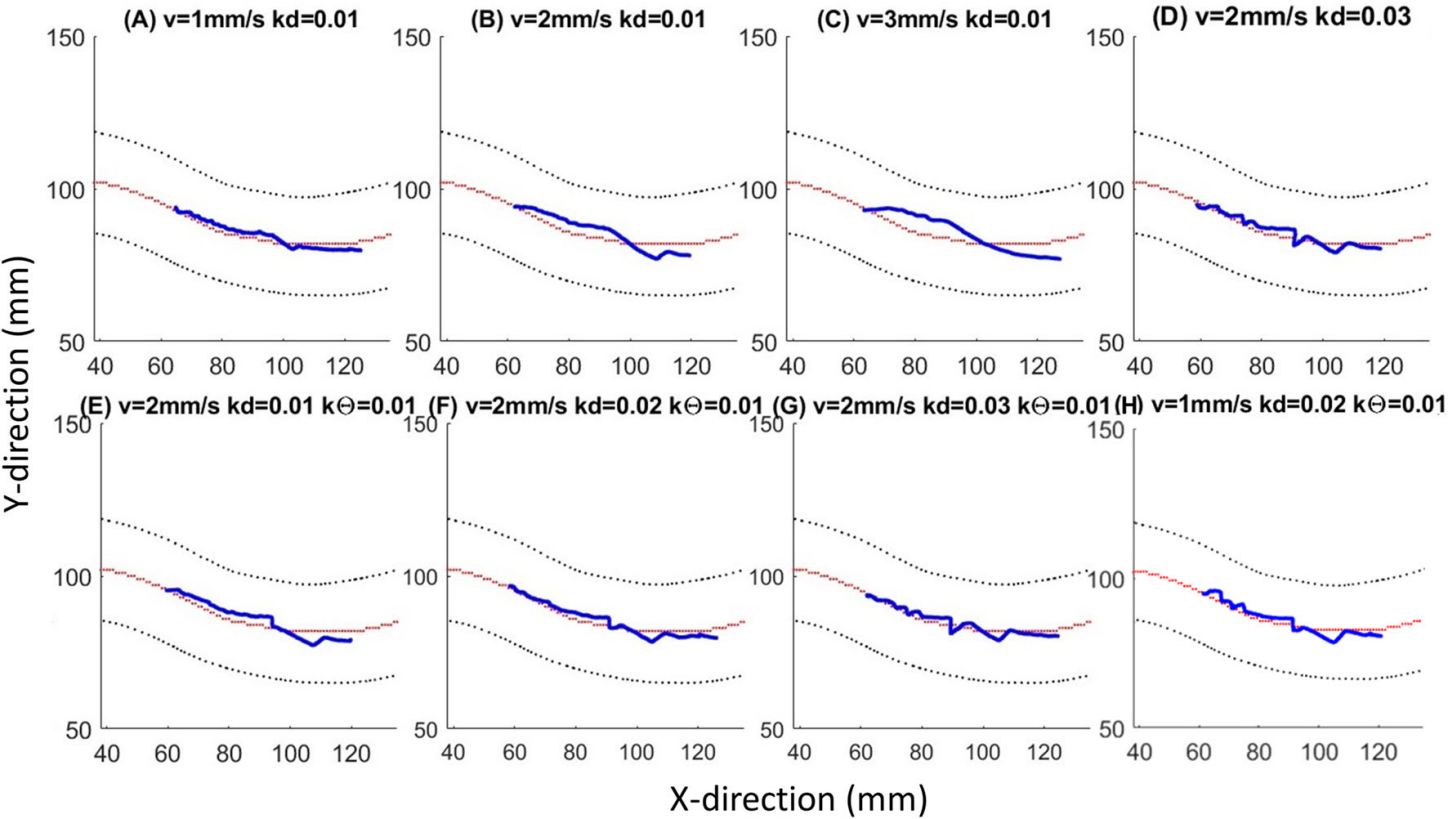
Results of the path tracking experiment: **A**–**H** Plots depict the tracking results with different settings, where the black lines represent the boundary of the vessel, the red lines represent the centerline of the vessel, and the blue lines are the recorded real trajectory from the camera data

The vessel phantom had the laser cutting boundary for the shape of the vessel and two acrylic planes for constraining motion in a plane (Fig. 6). The 3D anatomical model of the vessel was manually segmented and reconstructed using 3D slicer (Harvard University, National Institutes of Health), based on the CT scan images that we obtained from the hospital (Ospedale San Raffaele, Milan, Italy). Then, the 3D anatomy was projected in the coronal plane in Matlab (Mathworks, Massachusetts, USA), similar to the fluoroscopy image in the clinical procedure. The center line was extracted from the boundary of the vessel as the desired path (Fig. 7A). To reduce the friction resulting from the radial compression of the wooden boundary against the catheter body, a scaling factor of 1.5 to the vessel phantom was applied. The experiment was carried out in the first 60 mm section of the vessel phantom, which is the projection of the femoral vein and the iliac vein and the most tortuous part.

### Experimental protocols

To evaluate the accuracy of the control framework at each measured point *j*, we calculated the tracking error, $e_j$, which was the minimum Euclidean distance between the measured tip position of the catheter $\textbf{p}_{j} \left( x_{j},y_{j} \right)$ from the camera data and the pre-defined desired path $\textbf{p}_{i} \left( x_{i},y_{i} \right)$, which contains *I* points (Eq. 7).

7 $$\begin{aligned} e_j= & {} \min _{i, i\in \left\{ 1, 2, \dots , I \right\} } \left\| \textbf{p}_{j}-\textbf{p}_{i} \right\| \nonumber \\= & {} \min _{i, i\in \left\{ 1, 2, \dots , I \right\} } \sqrt{\left( x_{j}-x_{i}\right) ^{2}+\left( y_{j}-y_{i}\right) ^{2}} \end{aligned}$$

However, the distribution of errors along the entire path is not normal, because the level of challenge in navigating the catheter under various environmental constraints is different due to the insertion speed $\textbf{u}$, and the path geometry. To comprehensively evaluate tracking accuracy throughout the entire path, all the measurement was divided into ten equal sections, which contain *n* measured points. The associated median value (MED) in each section was calculated, which allowed us to intuitively compare the performance of the controller under different settings. Matlab (Mathworks, USA) was also used to calculate the maximum value (Max), and interquartile range (IQR). We examined the data set distribution with the Shapiro-Wilk normality test and applied corresponding descriptive statistics to compare different groups. In total, eight tests were carried out, and the velocity dependency was analyzed by setting the insertion speed $\textbf{u}$ at 1 mm/s, 2 mm/s, and 3 mm/s.

The proposed autonomous control framework was compared with a hybrid control system, which used a joystick controller (PS4, SONY Inc.) as the control element. In this system, the experiment participant was asked to maintain the tip of the catheter at the center of the vessel by controlling the steering DOF of the catheter with the joystick, while the catheter driver autonomously progressed the catheter at a constant insertion speed of 1 mm/s. After three tests, the most confident trajectory was selected as the hybrid control group to compare with the proposed autonomous control framework.

The controller was validated in both a free space and a constrained environment. In the first half of the path, there is no contact between the catheter and the boundary of the phantom (Fig. 7B). Then the catheter body collided with the upper boundary but navigation continues (Fig. 7C). The MED, Max, IQR was computed to evaluate the tracking accuracy in the region with and without environmental constraints.

## Experimental results and discussion

### Results

**Fig. 8:**
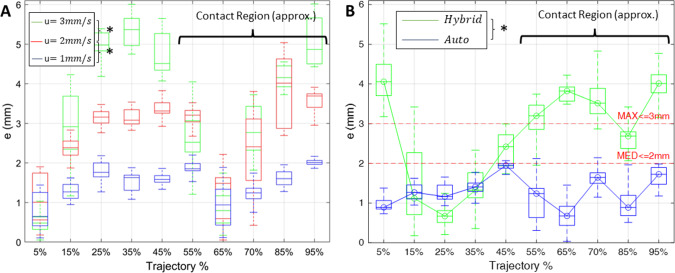
Experimental results in the box plots: **A** Experimental results show the performance of the controller at a different speed u in the range of 1–3 mm/s; **B** Tracking errors of the hybrid control method in green and the path tracking control in blue; $*p$-value <0.05

With the proposed controller, the tip of the robotic catheter successfully follows the center line of the vessel phantom. Figure 8A–H presents the trajectories recorded by the camera corresponding with different insertion speed and control gains, in which the real trajectories of the catheter tip in blue follow the centerline in red, and away from the black vessel border. The results of the Shapiro–Wilk normality test on the tracking error (p-value $< 0.05$) show that the error distributes abnormally on the path. The Kruskal–Wallis test shows that the insertion speed is significantly affecting the tracking performance (*p*-value $< 0.05$). The tracking results in the box plots indicate that the increase in the insertion speed will raise the tracking error because the robot doesn’t have enough time to respond and converge to the desired path. When the catheter is inserted at 1 mm/s, the controller has the most accurate performance with a MED along the entire path of 1.43 mm, and a Max of 2.19 mm (Fig. 9A). Compared with the hybrid control system, which has a MED value of 2.65 mm and a Max of 5.51 mm, the result in group H with the optimal control gain has a median value below 2 mm and a maximum error below 3 mm (Fig. 9B). The Mann–Whitney U test indicates a highly significant difference between the performances of those two methods (*p*-value $< 0.05$).

The contact between the catheter body and the vessel wall was identified at approximately $50\%$ of the trajectory where a salient drop was observed. To show the precision of the controller in both free space and the contact region, the MED, Max, and IQR in those regions were calculated and compared (Table 1). No significant difference was identified with the Mann–Whitney U test, indicating that the controller performed consistently in both free space and contact regions (*p*-value $> 0.05$), across different insertion speeds regarding groups H, G, and C.

### Discussion

We proposed a control framework to allow a safer and more autonomous insertion by avoiding contact between the catheter tip and the vessel wall. Our method achieves a convincing result and the insertion velocity has been considered in the control loop. In cardiovascular applications, the required precision that clinicians indicate as being acceptable is typically in the order of 1–3 mm [38, 39]. Our results from the in vitro studies showed that fidelity of the tracking could satisfy this requirement. Note that the test path does not cover the entire vessel which is 330 mm, because of the limited length of the catheter. A shortcoming of this work is the lack of adaptive methodology for regulating the insertion speed and the control gain factors. The insertion speed affects the performance of the controller and the final procedure time. The control gain factors are sensitive to the radius of the path and the contact region. Given these same wrenches, a Bayesian optimization approach may be used to choose those parameters for the patient-specific path.

**Table 1:** Performance comparison in free space and in contact region regarding the insertion speed

$\left\| \textbf{u} \right\|$(mm/s)	Free space (mm)			Contact region (mm)		
	MED	Max.	IQR	MED	Max.	IQR
1	1.07	2.10	0.68	1.53	2.20	0.67
2	1.35	2.42	0.65	0.91	3.00	0.83
3	3.43	6.00	1.67	3.46	6.01	2.92

In the future, the proposed controller can be extended in these two directions. 1) Path tracking with 3-D path: combing the rotation of the working plane could allow the catheter to achieve any points in the cartesian space. Furthermore, the working plane should be properly chosen to compensate for the gravity effect. 2) Adaptive control: The insertion velocity and the control gain factors could be regulated automatically based on the Bayesian optimization method to self-adapt different paths, which can reduce tracking errors and minimize the procedure time.

## Conclusion

In this work, we proved the feasibility of controlling a robotic catheter to track a given path with the proposed control framework. A tendon-driven steerable catheter was actuated and tested in vitro. The results suggest the potential for increasing the level of autonomy in robotic catheters to revolutionize transcatheter cardiology interventions. Compared with other model-based or model-less feedback control methods, our method achieves satisfying accuracy without the request of calibrating the intuitive parameters nor acquiring a training dataset. Moreover, nonholonomic motion planning and control have been extensively explored in robotics literature, allowing us to leverage a vast array of existing research in applying our model.
